# Insights Into CEST Contrast at 2 ppm in Enhancing and Nonenhancing Lesions From Glioma Patients Scanned at 7 T

**DOI:** 10.1002/nbm.70161

**Published:** 2025-10-16

**Authors:** Bárbara Schmitz‐Abecassis, Jeroen de Bresser, Linda Dirven, Martin J.B. Taphoorn, Matthias J. P. van Osch, Johan A. F. Koekkoek, Ece Ercan, the 7 T MRI Neuro‐Oncology consortium Monique Baas‐Thijssen

**Affiliations:** ^1^ Department of Radiology Leiden University Medical Center Leiden the Netherlands; ^2^ Medical Delta, South‐Holland the Netherlands; ^3^ Department of Neurology Leiden University Medical Center Leiden the Netherlands; ^4^ Department of Neurology Haaglanden Medical Center The Hague the Netherlands; ^5^ MR R&D, Clinical Science Philips Best the Netherlands

**Keywords:** CEST, glioma, guanidinium, Lorentzian fittings, MRI

## Abstract

**Introduction:**

Chemical exchange saturation transfer (CEST) has been demonstrated to provide a noninvasive opportunity to image gliomas. Preclinical ultrahigh‐field MRI studies have shown the value of the 2 ppm pool; however, in vivo studies in glioma patients are currently lacking. This study aimed to explore the 7 T MRI CEST contrast of the 2 ppm in gliomas and the tumor's different components.

**Methods:**

Twenty‐one glioma patients treated at two tertiary referral centers for brain tumors in the Netherlands were scanned. Regions of interest were defined as contrast‐enhancing (ce‐lesion), nonenhancing (NE‐lesion) tumor, and the contralateral normal‐appearing white matter (CL NAWM). Magnetization transfer ratio asymmetry (MTRasym), Lorentzian difference (LD), spillover and magnetization transfer‐corrected inverse difference (REX), and relaxation‐compensated (AREX) were calculated for all regions of interest.

**Results:**

The 2 ppm CEST pool signal between tumor regions and normal‐appearing tissue was found to be significantly different for all four ceST quantification methods (MTRasym *p* = 0.001; LD *p* < 0.001; REX *p* = 0.008; AREX *p* = 0.001). The CE and NE lesions showed significantly different 2 ppm pool CEST MTRasym (*p* = 0.034) and LD (*p* = 0.052). Significantly different 2 ppm CEST REX (*p* = 0.005) and AREX (*p* = 0.001) were found between the CL NAWM and the NE lesions.

**Conclusions:**

CEST 2 ppm pool contrast was distinctive between normal‐appearing white matter, enhancing and nonenhancing tumor lesions, independently of the metric used. These findings suggest that the CEST pool at 2 ppm provides a valuable noninvasive contrast for imaging gliomas.

## Graphical abstract

We investigated the 7 T MRI 2 ppm CEST pool contrast in (1.) high‐grade and (2.) low‐grade gliomas, finding significant differences between enhancing and nonenhancing lesions (3A,B), and between the healthy appearing white matter and nonenhancing lesions (3C,D). The 2 ppm CEST pool suggests valuable noninvasive contrast for glioma imaging.

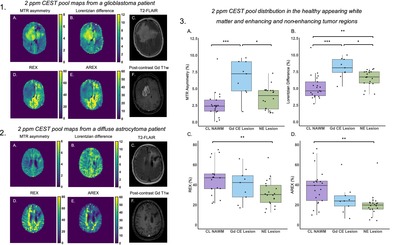

**Abbreviations**
APT: Amide proton transfer
AREX: Apparent exchange‐dependent relaxation
B_1_rms: B_1_ root mean squared
CE: Contrast‐Enhancing
CEST: Chemical Exchange Saturation Transfer
CL NAWM: Contralateral normal‐appearing white matter
DREAM: Dual refocusing echo acquisition mode
FLAIR: Fluid‐attenuated inversion recovery
FOV: Field of view
Gd CE: Gadolinium contrast‐enhancing
IDH: Isocitrate dehydrogenase
MTR: Magnetization transfer ratio
NE: Non‐Enhancing
NOE: Nuclear overhauser effect
ppm: Part per million
REX: Exchange‐dependent relaxation
*t*_sat_
: Total saturation
WASSR: Water saturation shift reference
WHO: World Health Organization

## 1 Introduction

Chemical exchange saturation transfer (CEST) is an MR modality that has been increasingly used to characterize brain tumors noninvasively. The majority of previous CEST work on brain tumors focused on the amide proton transfer (APT) pool at 3.5 ppm and nuclear overhauser (NOE) effects, and showed an increase in APT and a decrease in NOE from brain tumors compared to normal‐appearing tissue, respectively [1, 2, 3, 4]. On the other hand, only a few studies focused on the CEST pool at 2 ppm, although they showed its relevance for tumor differentiation. For example, preclinical work has shown that the CEST effect at 2 ppm is reduced in tumors compared to normal tissue [5]. This was especially observed upon tumor progression and increased aggressiveness [6]. The distribution of the 2 ppm CEST effect was also shown to correlate with creatine distribution in brain tumors [5]. Moreover, in clinical research, the CEST pool at 2 ppm, among others, was shown to help in the risk stratification of glioma patients based on tumor grade and molecular status [7]. An increased contrast of CEST at 2 ppm was found in the tumor compared to normal brain tissue [7]. The ratio of amides and amines has also been shown to be useful to help determine the tumor grade without the need for contrast agent‐enhanced imaging [8]. These examples illustrate how the 2 ppm CEST pool can be used to noninvasively characterize glioma, based on endogenous contrast. Such an opportunity could also be interesting as a future alternative to current invasive imaging techniques relying on exogenous contrast agents.

The use of gadolinium‐based contrast agents (GBCAs) for the acquisition of enhanced T_1_‐weighted images is currently the standard procedure for glioma patients, reflecting an increase in blood–brain barrier permeability [9]. However, some patients are allergic to such agents, making procedures more complicated. Moreover, there are concerns about gadolinium deposition in the brain, especially for patients undergoing extensive follow‐up imaging [10]. Because glioma patients need frequent radiological monitoring of potential tumor recurrence or progression, it becomes highly desirable to develop/use an endogenous imaging contrast, especially for patients with lower grade glioma who have a more prolonged survival. Finally, there is also a group of patients without tumor enhancement on their postcontrast T_1_‐weighted (T_1_w) scans [11]. This includes not only patients with lower grade gliomas, such as isocitrate dehydrogenase (IDH) mutant astrocytoma, but also patients with higher grade glioma, such as glioblastoma, IDH wild type [12, 13]. For these reasons, it becomes clinically relevant to explore different contrasts to noninvasively characterize glioma.

A few in vivo patient studies on the 2 ppm CEST pool have been performed on clinical systems, whereas most CEST studies on glioma patients have primarily focused on APT‐CEST. No clinical study has yet investigated how the CEST contrast at 2 ppm obtained using 7 T MRI differs between different tumor components (i.e., nonenhancing and enhancing lesions). Moreover, the quantification of the 2 ppm CEST pool is most often performed using MTR asymmetry. An evaluation of other quantification metrics such as Lorentzian difference (LD), the spillover and magnetization transfer corrected inverse difference (REX), and apparent exchange‐dependent relaxation (AREX), which have been previously used in APT‐CEST studies, is lacking. Given the higher spectral resolution at 7 T, we hypothesize it to be more specific to the pool resonating at 2 ppm. In this study, we aimed to investigate the CEST effects at 2 ppm in enhancing and nonenhancing lesions from glioma patients using several CEST quantification metrics (MTR Asymmetry, LD, REX, and AREX). Understanding how the contrast differs in these lesions compared to normal‐appearing white matter could possibly aid in the future with 1) the assessment of nonenhancing tumors, because these can also have poor prognosis, 2) understanding if this CEST contrast, in addition to the already established APT and NOE, could add value as a future alternative to the use of GBCAs.

## 2 Methods

### 2.1 Patient Inclusion

For this study, we prospectively included 21 patients from Leiden University Medical Center and Haaglanden Medical Center, the Netherlands, between March 2021 and May 2023. Adult patients with a histopathologically confirmed or highly suspected glioma, a Karnofsky Performance Status score ≥ 70, and no contraindications for MRI were eligible for inclusion. Patient characteristics are outlined in Table 1. The study adhered to the local institutional review board guidelines and approval. All patients gave written informed consent.

**TABLE 1:** Clinical characteristics of the participating glioma patients (*N* = 21).

Patient demographics		
Age ± standard deviation		57.9 ± 13.7
Female		11 (52%)
Male		10 (48%)
**Diagnosis**		
Glioblastoma, IDH‐wildtype		15 (71%)
Diffuse astrocytoma, IDH mutant		2 (10%)
Anaplastic astrocytoma, IDH‐mutant		2 (10%)
Oligodendroglioma, IDH‐mutant and 1p/19q co‐deleted		2 (10%)
**MGMT status**		
Methylated		7 (33%)
Unmethylated		8 (38%)
Unknown		6 (29%)
**Intervention**		
No intervention		4 (19%)
**Surgery**		17 (81%)
Partial resection		10 (48%)
Biopsy		7 (33%)
**Radiotherapy**		13 (62%)
Photon therapy		12 (57%)
Proton therapy		1 (5%)
Total dose	30 Gy	2 (10%)
	40 Gy	1 (5%)
	60 Gy	8 (38%)
**Adjuvant chemotherapy**		15 (71%)
Temozolomide		13 (62%)
Temozolomide and lomustine		1 (5%)
PCV (procarbazine, CCNU ([Lomustine], Vincristine)		1 (5%)
**Total daily use of dexamethasone**		
1 mg		4 (19%)
2 mg		1 (5%)
4 mg		1 (5%)
6 mg		1 (5%)

### 2.2 Imaging Data Acquisition

#### 2.2.1 Clinical Imaging

Clinical data were retrospectively collected from the hospital archives for all included patients. Patients were either scanned on a 3 T MR scanner (Philips Ingenia or Achieva, Philips Healthcare, Best, the Netherlands) or on a Siemens MAGNETOM Avanto 1.5 T scanner (Siemens, Erlangen, Germany). At 3 T, the 3D T_1_ postcontrast gadolinium enhanced scan was acquired with a 3D‐gradient‐echo readout and the following acquisition parameters: TR = 9.91 ms, TE = 4.67 ms, voxel size = 1 × 1 × 1 mm^3^, field of view (FOV) = 220 × 175 × 156 mm^3^, 0.3 mL per kg bodyweight of GBCA (gadoterate meglumine), and a total acquisition time of 2:57 min; the T_2_‐FLAIR was acquired with the following parameters: TR = 11,000 ms, TE = 125 ms, resolution = 0.4 × 0.4 × 5.5 mm^3^, FOV = 220 × 175 mm, and a total acquisition time of 2:12 min. At 1.5 T, the 3D T_1_‐postcontrast gadolinium enhanced scan was acquired with the following acquisition parameters: TR = 9 ms, TE = 2.38 ms, resolution = 0.9 × 0.9 × 0.9 mm^3^, FOV = 240 × 240 × 176 mm and 0.1 mL/kg of prebolus, and 20 mL of contrast agent (Dotarem 0.5 mmol/mL); the T_2_‐FLAIR was acquired with the following parameters: TR = 7500 ms, TE = 105 ms, voxel size = 0.4 × 0.4 × 5 mm^3^ and FOV = 230 × 230 × 144 mm^3^, and a total acquisition time of 2:08 min.

#### 2.2.2 CEST Imaging

CEST data were prospectively acquired at a whole‐body 7 T MRI scanner, Achieva Philips MRI scanner (Philips Healthcare, Best, the Netherlands) equipped with a dual‐transmit and a 32‐channel receiver head coil (Nova Medical Inc., Wilmington, MA, USA). The maximum achievable gradient strength and slew rate of the scanner are 40 mT/m and 200 T/m/s, respectively.

The acquisition protocol consisted of a short survey scan, a sensitivity encoding (SENSE) reference scan, a B_0_ map to be used to perform third‐order B_0_ shimming, a DREAM B_1_ map to correct for B_1_ inhomogeneities (TR = 8.0 ms, TE = 1.97 ms, FOV = 246 × 246 × 32 mm^3^ and a voxel size = 2 × 2 × 4 mm^3^, acquisition time: 08 s), a water saturation shift reference (WASSR) B_0_ map (TR = 3.3 ms, TE = 1.83 ms, FOV = 246 × 246 × 32 mm^3^ and a voxel size = 2 × 2 × 4 mm, acquisition time: 25 s) for postprocessing corrections and the CEST scan. A pulsed CEST preparation of 10 sinc‐gauss pulses of 50 ms duration and 50 ms interval (total saturation: 1000 ms) was followed by a gradient echo sequence with turbo field echo readout (TR = 3.3 ms, TE = 1.82 ms, FOV = 246 × 246 × × 32 mm^3^ and a voxel size = 2 × 2 × 4 mm^3^, acquisition time: 05:06 min). The B_1_ rms per pulse unit was 2.94 μT. In total, 22 frequencies were acquired with a step size of 136.4 Hz between −1500 Hz and 1500 Hz. Each volume took approximately 14 s. Finally, five scans were acquired with variable flip angles (2, 5, 7, 10 and 15°) which we used to estimate the T_1_ values for each patient (TR = 7.0 ms, TE = 1.3 ms, FOV = 246 × 246 × 32 mm and a voxel size = 2 × 2 × 4 mm, acquisition time: 2 s for each scan). The total acquisition time was 5:38 min.

### 2.3 Data Analysis

#### 2.3.1 CEST Analysis

Corrections for B_0_ inhomogeneities were performed using the WASSR scan according to the method described elsewhere [14]. The DREAM B_1_ map was used for correcting the AREX maps. The reference scan (M_0_ image) was acquired at a further offset of approximately 102 kHz to normalize the CEST images. The Z magnetization acquired was assessed over the frequency offsets for all voxels. In turn, 1‐S/S_0_ spectra were fitted to a 5‐pool Lorentzian model using the Levenberg–Marquardt algorithm. Fitting parameters can be found in the Supplementary Material in supplementary table S1. From the fitted data, we proceeded to calculate the LD, REX and the AREX metric as previously described [15]. The magnetization transfer ratio (MTR) asymmetry was calculated according to the following formula: $\text{MTRasym}=\frac{Z\left(-x\;\mathrm{ppm}\right)-Z\left(+x\;\mathrm{ppm}\right)\;}{Z\left(-x\;\mathrm{ppm}\right)}$ for the 2 and 3.5 ppm pools.

#### 2.3.2 Defining Tumor Areas of Interest

To compare the CEST contrast within the different tumor compartments, we delineated regions of interest (ROIs) within the gadolinium contrast‐enhancing tumor lesion (Gd ce lesion) and nonenhancing lesions (NE lesion), as well as an area in the contralateral normal‐appearing white matter (CL NAWM) for within‐patient comparison. The enhancing lesion segmentations were delineated on the postcontrast gadolinium enhanced T_1_w images, and only voxels that did not exhibit partial volume effects were considered. Therefore, we only included the Gd ce lesions of patients who had a tumor of at least 10 mm measured in the transverse plane (in the largest diameter); hence, lesions had to have at least 5 CEST voxels of 2 mm to be included. The NE lesions were delineated on the T_2_‐FLAIR, including the T_2_ hyperintense areas, which had to have a clear and solid T_2_ hyperintense area. We excluded the area of the Gd CE lesion and necrosis from the NE lesion segmentation. Lastly, a ROI within the CL NAWM was segmented within a region where the B_1_ was at least 85% of the maximum B_1_ to avoid having issues with B_1_ inhomogeneities for comparison.

#### 2.3.3 Statistical Analysis

To compare the CEST values between tumor lesions within each metric, a Kruskal–Wallis test was used, given the nonnormally distributed data. A post hoc test was then applied to assess which groups were significantly different between each other. The significance threshold was set at *p* ≤ 0.05. Statistical analysis was done using R version 4.1.2 (R Core Team 2021).

## 3 Results

**FIGURE 1:**
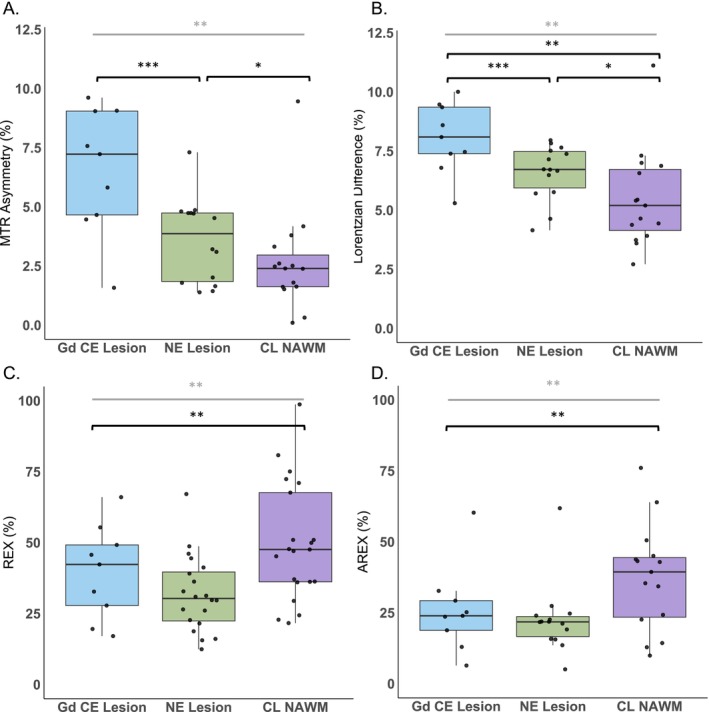
Scatter and box plots showing the 2 ppm CEST pool values for each patient and respective mean and standard deviations of the calculated A. MTR asymmetry, B. Lorentzian difference, C. *REX* , and D. AREX of the voxels included in the contralateral normal‐appearing white matter (CL NAWM), nonenhancing lesion (NE lesion), and gadolinium contrast enhanced lesion (Gd CE lesion) regions of interest. The significantly different results are illustrated with *, **, and *** for ≤ 0.05, ≤0.01, and ≤ 0.001, respectively; marked in grey and black for the Kruskal–Wallis test and post hoc test, respectively.

Figure 1 presents the average and standard deviation of CEST metric values calculated from the voxels of the CL NAWM, the NE lesion, and the Gd ce lesion. These data encompass all 21 patients; however, in one case, no NE lesion was present, and in 12 cases, no Gd ce lesions were observed. All quantification results can be found in the supplementary table S2.

MTR asymmetry of the 2 ppm ceST pool differed significantly between the different tumor lesions (*p* = 0.001). More specifically, the Gd CE lesions (6.6 ± 2.5) exhibited on average a significantly higher 2 ppm CEST contrast percentage values compared to the CL NAWM (2.6 ± 1.9, *p* = 0.002) and the NE lesion (3.6 ± 1.7, *p* = 0.034). Similarly, the LD also showed to be overall significantly different between lesions (*p* < 0.001), and had significantly higher 2 ppm LD values on the Gd ce lesion (8.1 ± 1.4), significantly higher than the CL NAWM (5.2 ± 1.8, p < 0.001), and the NE lesion (6.6 ± 1.2, *p* = 0.052). The latter two lesions also showed significantly different LD values (*p* = 0.004).

In Figure 1C, the REX measured at 2 ppm was overall significantly different between lesions (*p* = 0.008). This was specifically observed between the (CL NAWM = 50.0 ± 20.2) and the NE lesion (31.9 ± 13.0) (*p* = 0.005). The 2 ppm CEST contrast in the Gd CE lesion (39.5 ± 15.6) was on average lower than the CL NAWM and higher than the NE lesion, but not significantly different. For T_1_‐corrected AREX in Figure 1D, similar significant results were observed (*p* = 0.001). The CL NAWM (37.8 ± 17.5) had significantly higher 2 ppm CEST contrast compared to the NE lesion (20.1 ± 11.4) (*p* = 0.001). Contrarily, this was not the case for the Gd CE lesion (25.9 ± 14.3), which on average had a higher 2 ppm CEST contrast than the NE lesion, but not significantly.

**FIGURE 2:**
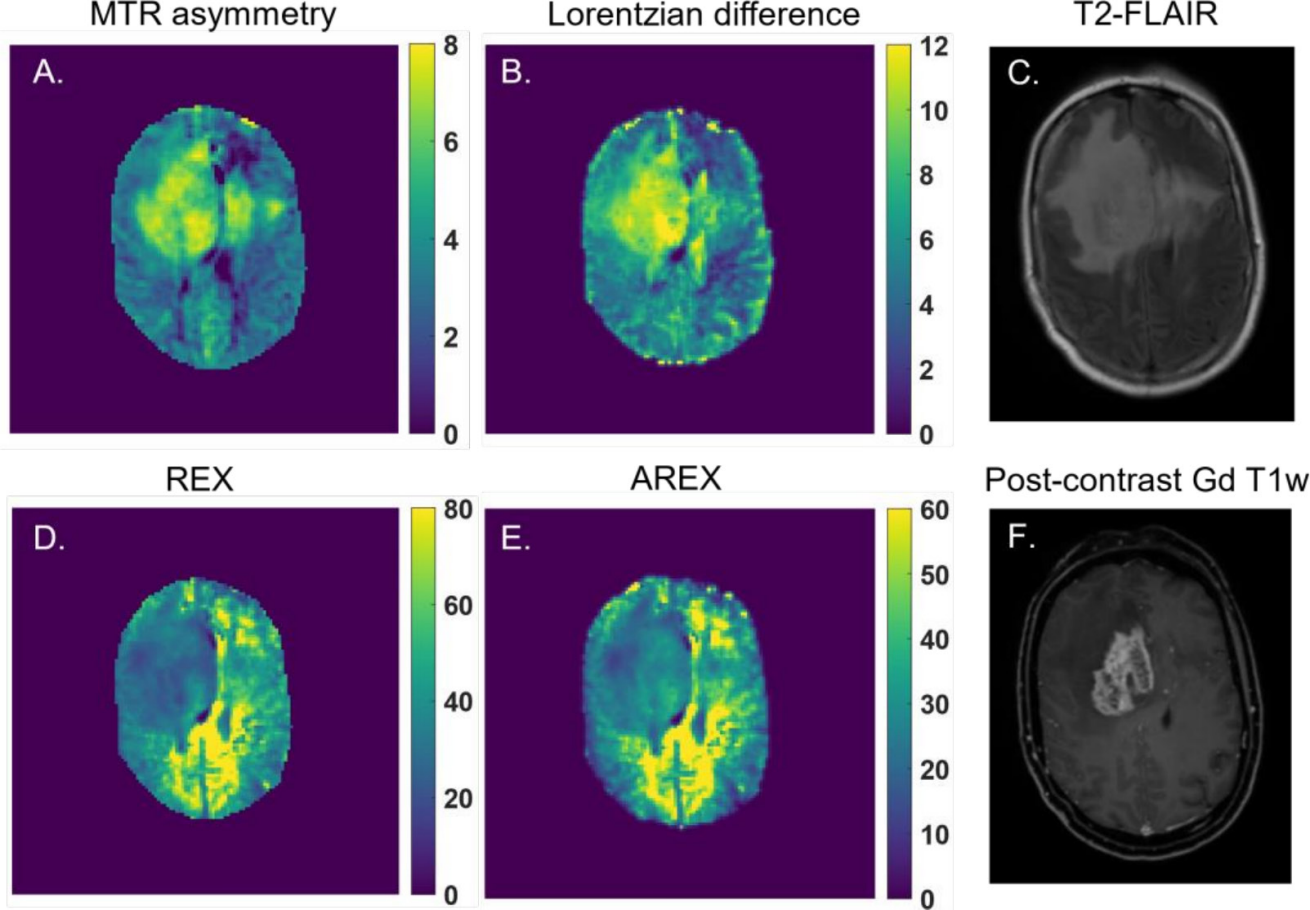
An example of a glioblastoma patient and the calculated 2 ppm CEST pool maps from A. MTR asymmetry (%), B. Lorentzian difference (%), D. *REX* (%), and E. AREX (%). In C. and F., the anatomical T_2_‐FLAIR and postcontrast Gd‐enhanced T_1_w are shown, respectively, illustrating where the tumor lesion can be found.

Figure 2 illustrates an example of a patient with glioblastoma, IDH wild type, World Health Organization (WHO) grade 4, displaying CEST maps generated from the different calculated CEST metrics from the CEST pool at 2 ppm. The respective T_2_‐FLAIR and postcontrast Gd T_1_w anatomical images are shown in Figure 2C and Figure 2F. It can be observed that the whole tumor area appears as hyperintense areas on the MTR asymmetry (Figure 2A) and LD (Figure 2B) maps. Conversely, the REX (Figure 2D) and AREX (Figure 2E) metrics display the tumor area as hypointense lesions.

**FIGURE 3:**
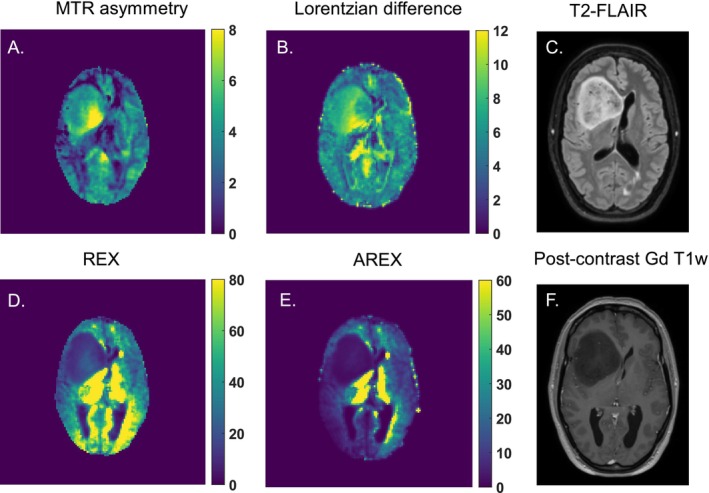
An example of an anaplastic astrocytoma (IDH mutant) and the calculated 2 ppm CEST pool maps from A. MTR asymmetry (%), B. Lorentzian difference (%), *D. REX* (%), and E. AREX (%). In C. and F., the anatomical T_2_‐FLAIR and postcontrast Gd‐enhanced T_1_w are shown, respectively, illustrating where the tumor lesion can be found.

Figure 3 presents an example of a patient with an anaplastic astrocytoma, IDH mutant, WHO grade 3, displaying the CEST maps for the CEST pool at 2 ppm. Although this tumor lacks an enhancing component (Figure 3F), a small hyperintense region (Figure 3C) is visible in the tumor area on the MTR asymmetry (Figure 3A) and LD (Figure 3B) maps. Conversely, the REX (Figure 3D) and AREX (Figure 3E) maps show a very hypointense tumor lesion, which corresponds well with the tumor area depicted in the anatomical images.

**FIGURE 4:**
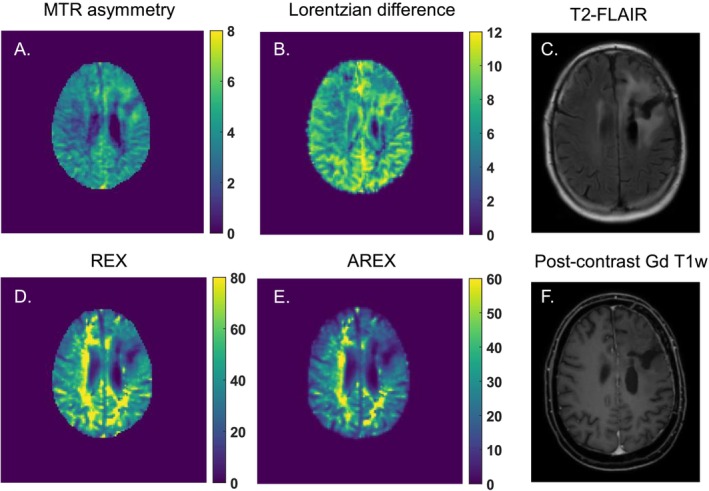
An example of a diffuse astrocytoma (IDH mutant) patient and the calculated 2 ppm CEST pool maps from A. MTR asymmetry (%), B. Lorentzian difference (%), *D. REX* (%), and E. AREX (%). In C. and F., the anatomical T_2_‐FLAIR and postcontrast Gd‐enhanced T_1_w are shown, respectively, illustrating where the tumor lesion can be found.

Figure 4 presents an example of a patient with astrocytoma, IDH mutant, WHO grade 2 with a nonenhancing tumor. The MTR asymmetry CEST map (Figure 4A) reveals a slight hyperintense area that aligns with the hyperintense tumor region on the T_2_‐FLAIR image (Figure 4C). This lesion also appears slightly hyperintense on the LD CEST map (Figure 4B). In contrast, the REX (Figure 4D) and AREX (Figure 4E) CEST maps display the same tumor lesion as hypointense.

**FIGURE 5:**
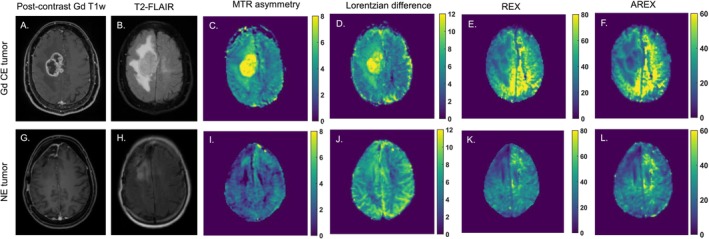
Two examples of enhancing and nonenhancing tumors on the upper and lower rows, respectively. A, D and G, H illustrate where the tumor lesions can be found on the postcontrast Gd T_1_w and T_2_‐FLAIR images, respectively. C–F and I–L show the resulting MTR asymmetry (%), Lorentzian difference, REX (%), and AREX (%) CEST map of the 2 ppm CEST pool for both cases.

Figure 5 displays a representative contrast‐enhancing tumor in the upper row and a nonenhancing tumor in the lower row. The enhancing tumor is a classic example of a glioblastoma, where the enhancing rim is clearly distinguished, surrounded by a nonenhancing area (Figure 5A,B). On both the CEST REX and AREX maps (Figure 5E,F), the enhancing rim appears as a hyperintense region encircling the necrotic core of the tumor, which shows up as hypointense. Conversely, on the MTR asymmetry and LD maps (Figure 5C,D), a hyperintense area is seen in the regions where the enhancing and necrotic areas are present. The surrounding nonenhancing areas are less hyperintense, which is in line with our results in Figure 1A and B. In contrast, the nonenhancing tumor in the lower row (Figure 5G,H) represents a lower‐grade tumor (anaplastic astrocytoma, IDH mutant, WHO grade 3) with a hypointense tumor area on the ceST REX and AREX maps (Figure 5K,L). The MTR asymmetry and LD maps show slightly higher 2 ppm CEST contrast in the tumor area (Figure 5I,J). The hypointense area on the REX and AREX maps in Figure 1K and L corresponds with the nonenhancing region seen on the T_2_‐FLAIR image and the surgical resection cavity, less evident on Figure 1I and J.

**FIGURE 6:**
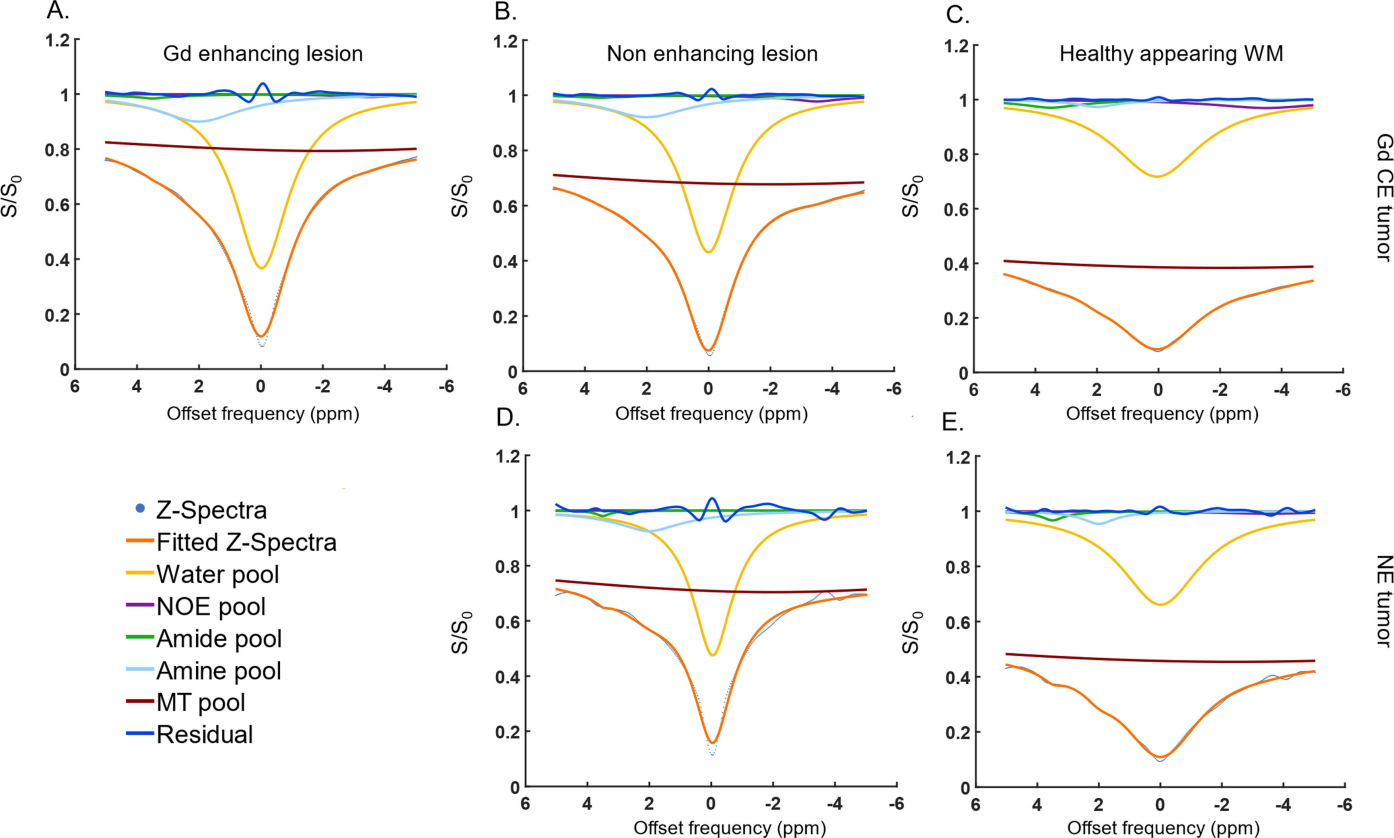
Two examples of 5‐pool Lorentzian fittings from a enhancing tumor, glioblastoma, IDH‐wildtype, (upper row) and nonenhancing tumor, anaplastic astrocytoma IDH‐mutant, (lower row). These graphs represent the average fittings of the voxels present in the A. gadolinium enhanced lesion, B., D. nonenhancing lesions and C., E. and contralateral normal‐appearing white matter.

Lastly, Figure 6 presents the Lorentzian fittings obtained from the average Z‐spectra of voxels within the Gd CE (Figure 6A), NE (Figure 6B–D), and CL NAWM (Figure 5C–E) segmentations. These fittings correspond to two representative tumors: an enhancing tumor in the upper row and a nonenhancing tumor in the lower row. As quantitatively demonstrated in Figure 1, the 2 ppm CEST pool values are higher in the CE and NE regions compared to the CL NAWM. Moreover, the broad Z‐spectra from the CL NAWM could be due to magnetization transfer (MT) effects.

## 4 Discussion

The goal of this study was to investigate the CEST contrast at 2 ppm in the different tumor areas of gliomas. To this end, we acquired the CEST images at 7 T MRI with a high B_1_rms accounting for the intermediate exchange rate of the CEST pool at 2 ppm [16, 17]. Hereafter, we applied a manual segmentation process to define the different tumor ROI. Finally, we quantified the CEST contrast utilizing different metrics already in use for glioma CEST imaging [18]. Our results showed that the 2 ppm CEST contrast differed between CE and NE lesions and CL NAWM depending on the metric used.

In this study, we evaluated for the first time the 7 T MRI CEST contrast of the 2 ppm CEST pool in the contrast‐enhancing and nonenhancing regions of gliomas. Although we did not investigate the source of this 2 ppm CEST contrast, one can speculate about its origins. Guanidinium protons resonating at this frequency can be found in metabolites such as creatine [19]. Creatine is known to be crucial for cellular metabolism. It provides phosphate from phospho‐creatine to synthesize adenosine triphosphate within the cells. Previous work has shown that oxygen consumption increases in the presence of creatine, illustrating why it is crucial for maintaining adequate cell energy production through aerobic metabolism [20]. Healthy cells rely mostly on oxygen for ATP production; however, tumors such as gliomas often experience a metabolic shift towards anaerobic metabolism [21].

We observed an overall increased 2 ppm CEST signal in CE and NE lesions compared to the CL NAWM. Previous animal work has shown similar results to ours, where the CEST contrast at 2 ppm, when fitting the Z‐spectra, was found to be significantly different in the tumor compared to the normal‐appearing tissue [5]. Our results in Figure 1 show that the contrast‐enhancing, nonenhancing, and normal‐appearing regions' 2 ppm CEST contrast significantly differed between each other. However, we could only find significant differences among all three regions while measuring the 2 ppm contrast with the LD. Although our MTR asymmetry results show a similar trend, most likely an increased standard deviation of the MTR asymmetry values in the nonenhancing lesions could have contributed to the fact that no significant difference was found between the CL NAWM and the NE lesion. Additionally, MTR asymmetry is sensitive to nonsymmetric contaminants from neighboring pools, nonspecific MT effects, and direct water saturation. These factors could have influenced the MTR asymmetry quantification, whereas their impact is less pronounced when calculating the LD. A similar explanation could account for the fact that for the REX and AREX results, we could only find a significant difference between the NE lesion and the CL NAWM. Figure 1C and D show a high standard deviation in all lesions, including a few outliers. When analyzing only the high‐grade patients, we found a significant overall difference between the MTR asymmetry and LD metrics. Although we cannot rule out potential differences between tumor grades, the limited statistical power arising from the limited sample size restricted us from evaluating subgroups with different tumor grades. In addition to the original analysis, when only the high‐grade patients were included, we found no significant differences between tumor regions for either REX or AREX. This is likely due to the relatively smaller effect sizes combined with the relatively large standard deviations (Supplementary Figure S1).

The fact that we do observe for at least one metric a significant difference in the 2 ppm CEST contrast between the enhancing and nonenhancing components in glioma is not surprising. These two lesions have been suggested to be metabolically distinct. For example, one study found that enhancing lesions have an elevated lactate and pyruvate level compared to the nonenhancing lesion, reflecting the glycolytic metabolic preference and can correlate with the tumor's malignancy grade [22]. Another possible explanation relies on the fact that enhancing areas, typically representing higher‐grade tumor activity, are metabolically more active. The increased energy requirements in order to meet cell proliferation demands could result in an increase in the presence of creatine in the cells. Nonenhancing lesions have less aggressive and infiltrative characteristics and could thus have relatively more preservation of creatine levels because the cells are metabolically less intense [23].

To support this, a previous study looked at the total creatine in WHO grade 2 gliomas. The authors found that normalized total creatine levels were a significant predictor for tumor progression and malignant transformation in lower‐grade gliomas [24]. They concluded that, in general, low‐grade gliomas with a relative total creatine level below 1.0 may have a longer progression‐free survival and a delayed risk of malignant transformation compared to cases with higher total creatine levels.

A more recent study investigated longitudinal changes in low‐grade gliomas using multiparametric MRI and applied magnetic resonance spectroscopy imaging to monitor metabolic changes, specifically the creatine‐to‐NAA ratio (CRNI). Notably, CRNI values were found to be higher in astrocytomas that showed progression compared to stable cases; however, this pattern was not observed in oligodendrogliomas. The authors suggest that changes in creatine levels may be specific to WHO glioma subtypes [25]. Another study reported that glioblastomas had lower creatine levels than astrocytomas, WHO grade 2 or 3 [26]. In poorly perfused regions with abnormal pH, high‐grade gliomas also showed reduced creatine and elevated lactate levels. Tumor areas with increased cerebral blood volume demonstrated higher creatine levels, likely due to the initial rise in energy demands associated with new blood vessel formation [25]. Additionally, another study found decreased creatine in grade 3 tumors overall, although areas of higher metabolism exhibited elevated creatine. An increase in creatine may be an early indicator of cellular energy stress, preceding the shift to anaerobic respiration and subsequent lactate accumulation [25]. A more recent study looked at different metabolite concentrations while combining low‐ and high‐grade glioma MRS imaging data at 7 T. Similarly to our study, by including both low‐ and high‐grade gliomas, the authors observed an overall decrease in total creatine in the tumor regions, although a few cases showed an increase in total creatine [23].

CEST contrast at 2 ppm is known to have a substantial contribution from guanidinium protons [27]. A recent study investigated the origins of the CEST contrast at 2 ppm in the rat brain [28]. The authors concluded that the contribution from guanidinium protons in amino acids cannot be excluded when interpreting the CEST contrast at 2 ppm in the rat brain. Although confirmation of these results in humans still needs to follow, these results show the importance of being cautious regarding interpreting the origins of the 2 ppm CEST contrast in the brain. More recent studies in the human brain have also investigated the contribution of the guanidinium pool to the CEST contrast at 2 ppm. They concluded that CEST contrast at 2 ppm is an interesting technique for creatine mapping of the brain and showed guanidinium CEST mapping in a brain tumor patient at 3 T [29, 30]. Because amide protons are known to be present in glioma and guanidinium protons from proteins contribute to the contrast at 2 ppm, it would be interesting to explore how much of the 2 ppm CEST contrast in tumors comes from guanidinium in proteins versus guanidinium in creatine.

Given CEST's limited specificity, the environmental conditions surrounding the metabolite or protein of interest are critical in determining the optimal parameters for accurate detection. CEST contrast is influenced not only by temperature but also by pH [31]. For instance, the extravascular extracellular tumor microenvironment is often acidic due to lactate efflux, while the intracellular environment has a high basic pH [32]. In such a high pH environment, guanidinium protons at 2 ppm undergo rapid exchange. To maximize sensitivity, the CEST sequence used in our study was designed with a high saturation power. Because guanidinium protons under the same acidic conditions are known to exchange at an intermediate rate [33] (compared to amines), we hypothesize that the CEST contrast we observed is primarily driven by guanidinium protons.

While further experiments are needed to confirm the exact origin of the contrast, our results highlight the possibilities of using the 2 ppm CEST pool in differentiating tumor components. Although we did not correct for pH in our analysis, the distinct contrasts observed across various tumor regions suggest that the pH of the tumor microenvironment may play a significant role in these findings. Previous studies have explored pH‐weighted imaging of the 3 ppm amine pool using CEST in glioblastomas [34]. One key finding was that an increase in the amine CEST effect occurred only in low pH environments, demonstrating its potential as a noninvasive tool for detecting acidic regions. This increase in contrast was predominantly observed in the more active and malignant tumor areas. Similarly, we observed an increase in CEST contrast in the enhancing regions of the tumors we studied, with a comparable MTR asymmetry.

In our study, we incorporated various CEST metrics to evaluate how the results might vary across them. Lorentzian fitting is theoretically more accurate as it accounts for relaxation effects. AREX further improves reliability by incorporating correction for T_1_‐effects, allowing for more precise quantification. However, its accuracy depends on precise T_1_ mapping and sufficient SNR for robust spectral analysis. Similarly, Lorentzian line shape fitting allows the separation of different CEST effects, including direct water saturation and MT. The main challenge, however, is acquiring high‐quality data to effectively distinguish the various exchange pools. Contrarily, MTR asymmetry is the most widespread used quantification metric due to its simplicity, which includes a straightforward reporting on the different CEST pools. However, it is prone to contamination from competing effects, including MT and direct water saturation. Figure 1 illustrates that our findings remained largely consistent, although it should be kept in mind that the markers of Figure 1C and D represent an inverse metric compared to Figure 1A and B. The primary difference observed was that enhancing lesions exhibited higher values than nonenhancing lesions for MTR Asymmetry and LD metrics compared to REX and AREX. The difference may be attributed to the fact that both REX and AREX metrics correct for MT and spillover effects. MTR asymmetry and the LD do not. Consequently, eliminating these confounding effects could provide a more accurate representation of the CEST contrast at 2 ppm. Similarly, we observed a larger standard deviation in the LD, REX, and AREX results, specifically for the CL NAWM. These differences could be present due to partial volume effects in the chosen ROIs due to the voxel size, despite our efforts to minimize the partial volume effects while drawing the ROIs. Secondly, the CL NAWM ROIs were drawn on the same slice as the tumor. Because some tumors were located in lower brain regions, where magnetic field inhomogeneities are more pronounced, this may have affected T_1_ mapping. Lastly, and perhaps most importantly, the concentration of guanidinium protons in white matter is relatively lower compared to tumors. Due to this lower concentration, detecting a sufficient signal becomes more challenging, making the fitting process more susceptible to noise.

However, due to our limited patient sample, these findings need to be validated in a larger cohort to potentially attenuate outliers and, in some cases, large standard deviations, which could have impacted our statistical conclusions. Our results suggest that the 2 ppm CEST contrast differs between enhancing, nonenhancing lesions, and normal‐appearing tissue. Determining the optimal metric for this purpose lies beyond the scope of our study, as the primary aim was to explore whether the results would vary depending on the metric used to quantify the CEST contrast of interest.

Our study has several strengths, including a relatively large cohort of glioma patients with prospectively acquired CEST data at 7 T MRI. Given the prospective nature, we were able to optimize and implement a CEST protocol specific for fast exchanging amines (i.e., higher B_1_ rms), which is different from the protocol used for slow exchanging CEST pools such as APT and NOE. Additionally, clinical MR scans were available, serving as the gold standard for imaging comparison. Another key strength is the use of different CEST metrics to measure CEST contrast in this patient group for the first time.

However, there are also some limitations to consider. First, we included a somewhat heterogeneous group of glioma patients, both pretreatment and posttreatment. As a result, we cannot entirely rule out the potential influence of treatment procedures on the observed CEST contrast. Furthermore, because of this broad patient group, the differences in 2 ppm CEST contrast between high‐ and low‐grade tumors remain unexplored. Second, we used a linear B_1_ correction method. While other correction methods have demonstrated superior performance, they were not feasible for this study due to their time‐consuming nature during data acquisition. Thirdly, our saturation pulses were interleaved with 50 ms gaps. The interpulse delays are included to adhere to specific absorption rate (SAR) limits and the RF amplifier requirements of the MR scanner used [16]. Ideally, we would have employed shorter interpulse delays and longer or more saturation pulses, which would be more desirable to enhance the contrast between intermediate and fast exchanging pools. Because of these limitations, it could be that there could be some contamination from slow exchanging amides in our signals. Lastly, despite optimizing our protocol for the CEST pool at 2 ppm, we cannot definitively determine the precise origin of the CEST contrast, as MRS measurements were not included.

These preliminary results indicate promising applications for 2 ppm CEST in glioma imaging. Future research could focus on expanding this methodology to different glioma subgroups, including patients with different clinical symptoms (e.g., epilepsy), allowing for a deeper understanding of how the CEST pool at 2 ppm contrast varies across disease stages. Additionally, studying groups exclusively pretreatment or posttreatment could help clarify the impact of treatment on the 2 ppm CEST contrast. Finally, increasing the sample size would enable a more robust evaluation of potential statistical differences between groups.

In conclusion, our study demonstrated a significantly distinctive 2 ppm CEST contrast between normal‐appearing white matter, enhancing, and nonenhancing tumor lesions in glioma patients. These findings suggest that the CEST pool at 2 ppm may provide valuable noninvasive contrast for glioma evaluation, though further confirmation in a larger cohort is necessary.

## Supporting information


**Table S1:** Fitting parameters used for Lorentzian fittings.
**Table S2:** CEST (%) values per quantification metric from each tumor lesion. Values are displayed for all patients and respective diagnosis.
**Figure S1:** Scatter and box plots showing the 2 ppm pool values for the Glioblastoma IDH‐wildtype patients and respective mean and standard deviations of the calculated A. MTR asymmetry, B. Lorentzian difference, *C. REX* and D. AREX of the voxels included in the contralateral normal‐appearing white matter (CL NAWM), nonenhancing lesion (NE lesion) and gadolinium contrast enhanced lesion (Gd CE lesion) regions of interest. The significantly different results are illustrated with * and ** for ≤ 0.05 and ≤ 0.01, respectively; marked in grey and black for the Kruskal–Wallis test and post hoc test, respectively.

## Data Availability

Data will be made available upon reasonable request.
