# Supplementary material for: Insights Into CEST Contrast at 2 ppm in Enhancing and Nonenhancing Lesions From Glioma Patients Scanned at 7 T
Source: NMR Biomed. 2025 Oct 16;38(12):e70161. doi: 10.1002/nbm.70161 (PMC12531428; doi:10.1002/nbm.70161)
Supplement: Supplementary file 1 — Table S1: Fitting parameters used for Lorentzian fittings. Table S2: CEST (%) values per quantification metric from each tumor lesion. Values are displayed for all patients and respective diagnosis. Figure S1: Scatter and box plots showing the 2 ppm pool values for the Glioblastoma IDH‐wildtype patients and respective mean and standard deviations of the calculated A. MTR asymmetry, B. Lorentzian difference, C. REX and D. AREX of the voxels included in the contralateral normal‐appearing white matter (CL NAWM), nonenhancing lesion (NE lesion) and gadolinium contrast enhanced lesion (Gd CE lesion) regions of interest. The significantly different results are illustrated with * and ** for ≤ 0.05 and ≤ 0.01, respectively; marked in grey and black for the Kruskal–Wallis test and post hoc test, respectively. [file NBM-38-e70161-s001.docx]

**Supplementary Material**

Supplementary Table S1. Fitting parameters used for Lorentzian fittings.

| **Pool** | **Start** | **Lower** | **Upper** |
| --- | --- | --- | --- |
| Amplitude: Water | 0.9 | 0.02 | 1 |
| Width: Water | 1.4 | 0.3 | 10 |
| Frequency: Water | 0 | -0.5 | 0.5 |
| Amplitude: MT | 0.1 | 0 | 1 |
| Width: MT | 25 | 0 | 150 |
| Frequency: MT | 0 | -4 | -2 |
| Amplitude: NOE | 0.02 | 0 | 0.4 |
| Width: NOE | 3 | 1 | 5 |
| Frequency: NOE | -3.5 | -3.5 | -3.5 |
| Amplitude: APT | 0.025 | 0 | 0.2 |
| Width: APT | 0.5 | 0.4 | 3 |
| Frequency: APT | 3.5 | 3.5 | 3.5 |
| Amplitude: Amines | 0.01 | 0 | 0.2 |
| Width: Amines | 1.5 | 1 | 3.5 |
| Frequency: Amines | 2 | 2 | 2 |

|  |  |  | **MTR asymmetry (%)** | | | **Lorentzian Difference (%)** | | | **REX (%)** | | | | **AREX (%)** | | |
| --- | --- | --- | --- | --- | --- | --- | --- | --- | --- | --- | --- | --- | --- | --- | --- |
| **Patients** | **Diagnosis** | **WHO grading** | **Gd CE Lesion** | **NE Lesion** | **CL NAWM** | **Gd CE Lesion** | **NE Lesion** | **CL NAWM** | **Gd CE Lesion** | **NE Lesion** | | **CL**  **NAWM** | **Gd CE Lesion** | **NE Lesion** | **CL NAWM** |
| 1 | Glioblastoma, IDH-wildtype | Grade 4 | 9,1 | 1,4 | 4,2 | 9,4 | 7,6 | 4,6 | 55,4 | 44,5 | | 75,1 | 32,7 | 27,4 | 50,5 |
| 2 | Glioblastoma, IDH-wildtype | Grade 4 | 9,1 | 1,4 | 1,6 | 7,5 | 7,5 | 4,4 | 45,7 | 39,1 | | 71,0 | 23,6 | 24,0 | 76,1 |
| 3 | Glioblastoma, IDH-wildtype | Grade 4 |  | 1,7 | 1,8 |  | 4,2 | 3,6 |  | 18,8 | | 36,2 |  | 21,9 | 39,4 |
| 4 | Glioblastoma, IDH-wildtype | Grade 4 |  | 4,8 | 1,6 |  | 6,7 | 5,2 |  | 48,7 | | 36,0 |  | 22,6 | 22,6 |
| 5 | Glioblastoma, IDH-wildtype | Grade 4 | 4,5 |  | 2,5 | 7,4 |  | 5,4 | 32,7 |  | | 67,6 | 18,8 |  | 42,9 |
| 6 | Glioblastoma, IDH-wildtype | Grade 4 | 9,6 | 7,3 | -0,3 | 8,1 | 6,5 | 7,0 | 19,6 | 22,7 | | 36,3 | 13,0 | 15,8 | 34,3 |
| 7 | Glioblastoma, IDH-wildtype | Grade 4 |  | 3,2 | -0,1 |  | 5,7 | 3,9 |  | 30,9 | | 21,7 |  | 13,6 | 14,3 |
| 8 | Glioblastoma, IDH-wildtype | Grade 4 | 4,7 | 4,7 | 3,3 | 5,3 | 5,8 | 4,4 | 66,0 | 67,1 | | 45,2 | 60,3 | 61,8 | 45,0 |
| 9 | Glioblastoma, IDH-wildtype | Grade 4 | 7,2 | 4,5 | 3,8 | 8,6 | 7,8 | 7,3 | 42,3 | 26,5 | | 37,1 | 25,2 | 15,6 | 24,3 |
| 10 | Glioblastoma, IDH-wildtype | Grade 4 | 5,8 | 4,7 | 1,5 | 10,0 | 8,0 | 2,7 | 27,8 | 29,7 | | 51,0 | 29,3 | 21,9 | 43,9 |
| 11 | Anaplastic astrocytoma, IDH-mutant | Grade 3 |  | 2,1 | 2,3 |  | 7,5 | 4,6 |  | 21,5 | | 49,9 |  | 19,4 | 42,6 |
| 12 | Oligodendroglioma (suspected) | Grade 2 |  | 3,5 | 2,5 |  | 6,0 | 3,6 |  | 41,3 | | 72,4 |  | 17,2 | 41,7 |
| 13 | Glioblastoma, IDH-wildtype | Grade 4 |  | 3,1 | 2,4 |  | 6,7 | 5,4 |  | 26,1 | | 51,0 |  | 21,2 | 43,2 |
| 14 | Glioblastoma, IDH-wildtype | Grade 4 |  | 4,9 | 2,5 |  | 6,7 | 6,9 |  | 46,1 | | 22,9 |  | 21,7 | 12,9 |
| 15 | Glioblastoma, IDH-wildtype | Grade 4 | 7,6 | 4,7 | 2,6 | 9,5 | 7,4 | 3,7 | 49,2 | 36,3 | | 80,8 | 23,9 | 24,7 | 63,9 |
| 16 | Diffuse Astrocytoma, IDH mutant | Grade 2 |  | 1,4 | 2,0 |  | 4,1 | 4,2 |  | 12,4 | | 29,5 |  | 7,4 | 21,6 |
| 17 | Diffuse Astrocytoma, IDH mutant | Grade 2 |  | 4,4 | 3,4 |  | 7,5 | 6,0 |  | 32,8 | | 47,6 |  | 16,8 | 30,3 |
| 18 | Glioblastoma, IDH-wildtype | Grade 4 |  | 2,0 | 9,5 |  | 4,6 | 11,1 |  | 31,3 | | 47,0 |  | 19,1 | 35,4 |
| 19 | Anaplastic astrocytoma, IDH-mutant | Grade 3 |  | 4,8 | 0,7 |  | 7,9 | 3,8 |  | 16,1 | | 47,7 |  | 4,4 | 27,3 |
| 20 | Oligodendroglioma, IDH-mutant and 1p/19q co deleted | Grade 2 |  | 6,0 | 4,4 |  | 5,8 | 5,1 |  | 29,7 | | 98,7 |  | 20,3 | 71,5 |
| 21 | Glioblastoma, IDH-wildtype | Grade 4 | 1,6 | 1,8 | 2,4 | 6,8 | 7,2 | 6,6 | 17,1 | | 15,6 | 24,4 | 6,4 | 5,1 | 9,9 |

Supplementary Table S2. CEST (%) values per quantification metric from each tumor lesion. Values are displayed for all patients and respective diagnosis.


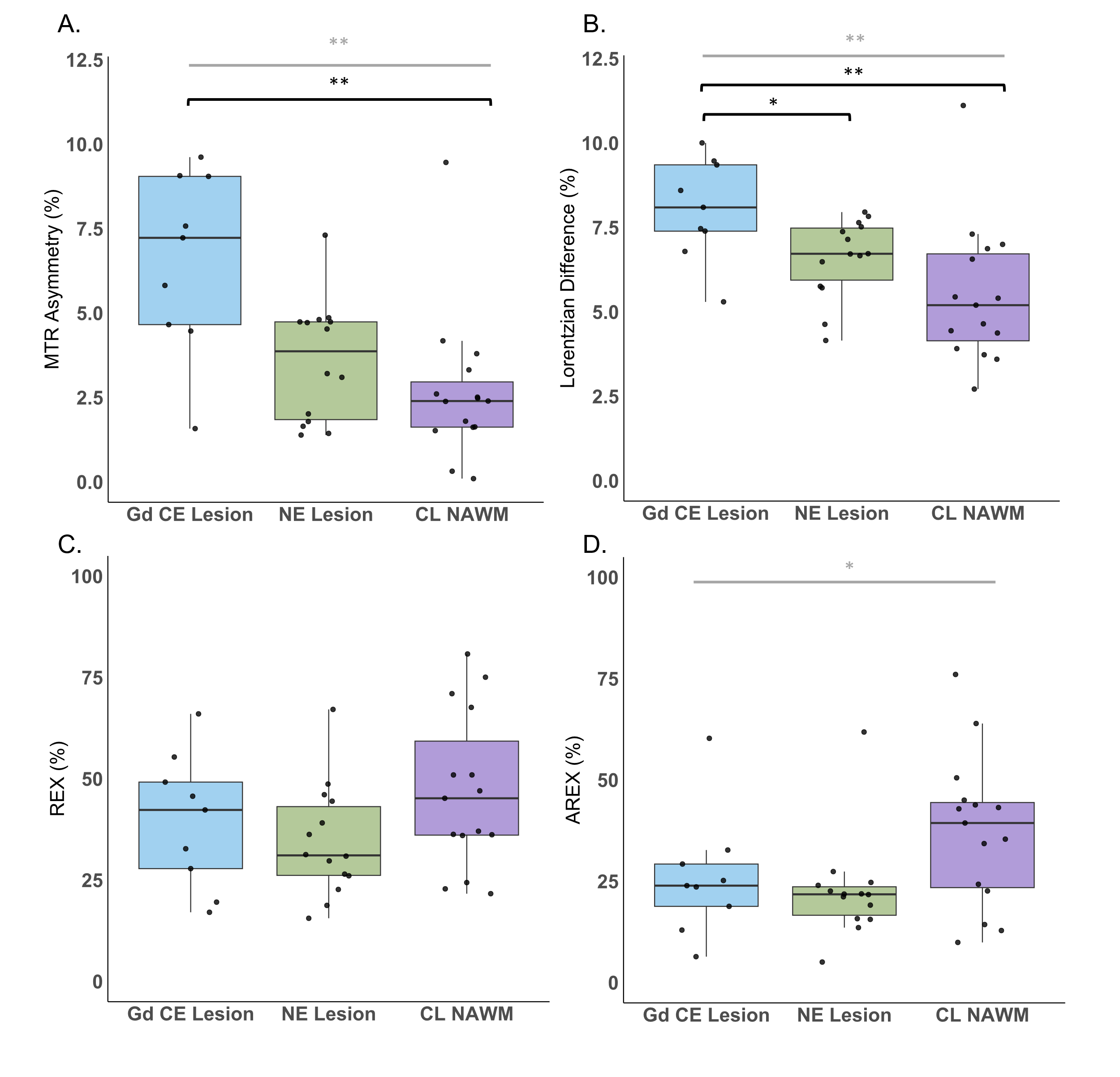
Supplementary Figure

Figure S1. Scatter and box plots showing the 2 ppm pool values for the Glioblastoma IDH-wildtype patients and respective mean and standard deviations of the calculated A. MTR asymmetry, B. Lorentzian difference, C. REX and D. AREX of the voxels included in the contralateral normal appearing white matter (CL NAWM), non-enhancing lesion (NE lesion) and gadolinium contrast enhanced lesion (Gd CE lesion) regions of interest. The significantly different results are illustrated with * and ** for ≤ 0.05 and ≤0.01, respectively; marked in grey and black for the Kruskal Wallis test and post-hoc test, respectively.

**
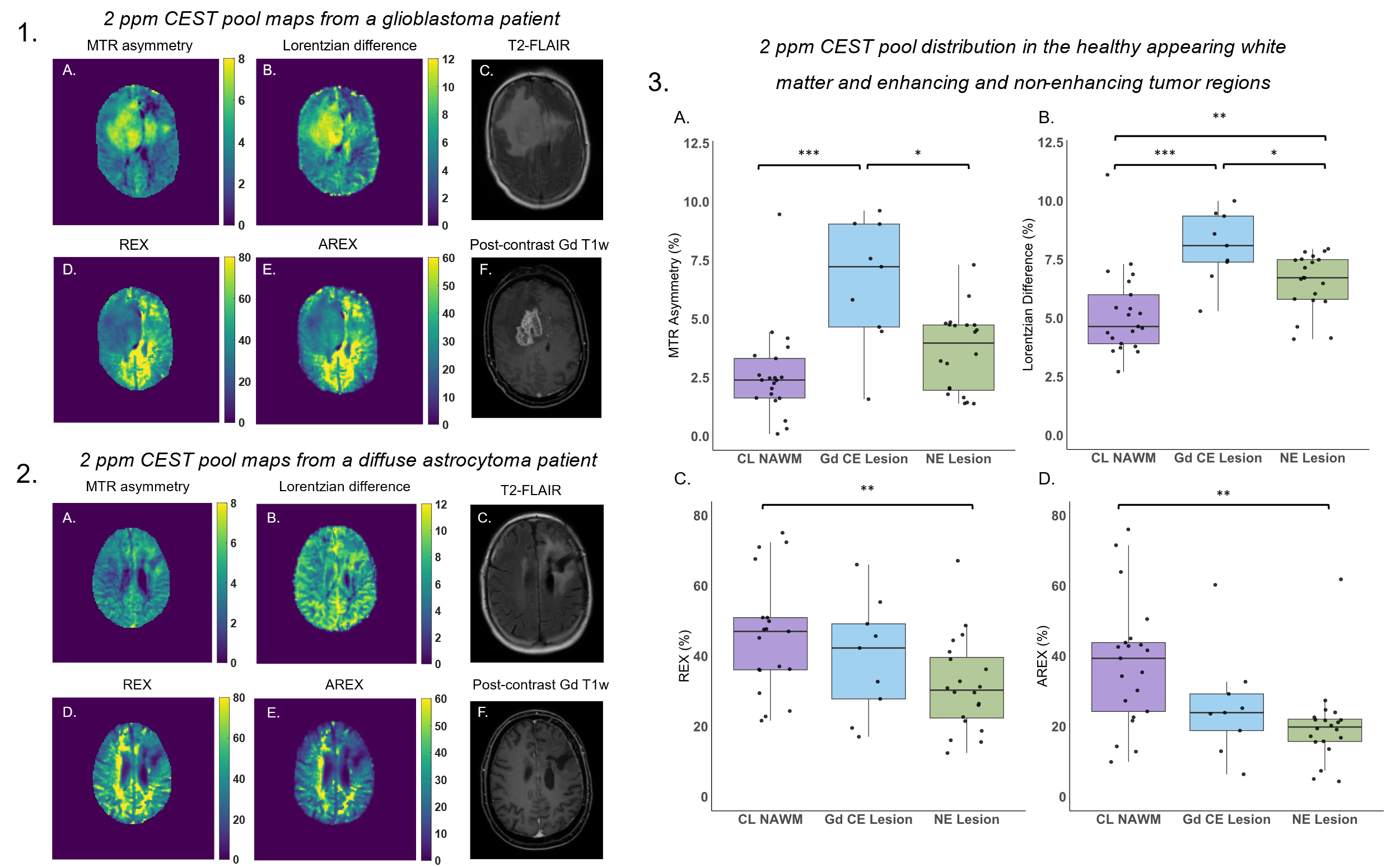
Graphical abstract**

We investigated the 7T MRI 2 ppm CEST pool contrast in (1.) high-grade and (2.) low-grade gliomas, finding significant differences between enhancing and non-enhancing lesions (3A-B), and between the healthy appearing white matter and non-enhancing lesions (3C-D.).The 2 ppm CEST pool suggests valuable non-invasive contrast for glioma imaging.
